# Spindle Activity Orchestrates Plasticity during Development and Sleep

**DOI:** 10.1155/2016/5787423

**Published:** 2016-05-16

**Authors:** Christoph Lindemann, Joachim Ahlbeck, Sebastian H. Bitzenhofer, Ileana L. Hanganu-Opatz

**Affiliations:** Developmental Neurophysiology, Institute of Neuroanatomy, University Medical Center Hamburg-Eppendorf, 20251 Hamburg, Germany

## Abstract

Spindle oscillations have been described during early brain development and in the adult brain. Besides similarities in temporal patterns and involved brain areas, neonatal spindle bursts (NSBs) and adult sleep spindles (ASSs) show differences in their occurrence, spatial distribution, and underlying mechanisms. While NSBs have been proposed to coordinate the refinement of the maturating neuronal network, ASSs are associated with the implementation of acquired information within existing networks. Along with these functional differences, separate synaptic plasticity mechanisms seem to be recruited. Here, we review the generation of spindle oscillations in the developing and adult brain and discuss possible implications of their differences for synaptic plasticity. The first part of the review is dedicated to the generation and function of ASSs with a particular focus on their role in healthy and impaired neuronal networks. The second part overviews the present knowledge of spindle activity during development and the ability of NSBs to organize immature circuits. Studies linking abnormal maturation of brain wiring with neurological and neuropsychiatric disorders highlight the importance to better elucidate neonatal plasticity rules in future research.

## 1. Introduction

Information processing within the brain critically depends on rhythmic oscillatory activity that synchronizes neuronal networks. Synchronization leads to local and global coupling of network elements and times neuronal firing. By these means, it enables the precise selection of relevant information. Depending on the brain state and the timing of convergent inputs, plastic changes up- or downgrade the importance of new information from the environment [1, 2]. Consolidation of the collected information in long-term memory during sleep guarantees a timely reaction to environmental changes and promotes survival [3–5].

Spindle oscillations are typical representatives of rhythmic network activity that have been monitored in electroencephalographic (EEG) recordings both during early development and at adulthood [6–10]. While the underlying mechanisms of ASSs have been extensively investigated in the past, aiming to identify their role for consolidation of memories [4, 5, 11], NSB-related mechanisms are still largely unresolved and their function remains rather blurry. Only recent experimental evidence indicated that NSBs do not represent a by-product of maturating neuronal networks but important elements for their refinement [12–16]. This review summarizes the different aspects of ASS and NSB plasticity.

## 2. Adult Sleep Spindles

ASSs are recurrent, short lasting network oscillations (0.5–3 s) characteristic for nonrapid eye movement (NREM) sleep. They have a waxing and waning waveform with the main frequency ranging from 9 to 15 Hz [6, 11]. Spontaneous ASSs synchronize large cortical areas, following defined patterns of spatial distribution that have been monitored in humans both by EEG and magnetoencephalogram (MEG) [17–19]. This distribution depends on different factors, such as spindle peak frequency (slow or fast), sleep stage period (early or late), and age of the investigated person [20–23]. According to their waveform properties and cortical distribution, two entities of ASSs have been distinguished. On the one hand, prominent slow ASSs (9–13 Hz) emerge as product of spindle generators located in frontal brain regions. On the other hand, low amplitude fast ASSs (13–15 Hz) originate from the thalamic reticular nucleus (TRN) and spread over the whole cortex with strongest occurrence in central and parietal areas [23–27]. However, the exact origin of slow and fast ASSs and whether they share the same generators is still a matter of debate [18, 27–30]. Support for separate underlying generators comes from differential pharmacological modulation of slow and fast ASSs [24].

In contrast to humans, mice show no difference in the frequencies between frontal and centroparietal spindles. Still, ASSs can be divided into three different types based on their anterior, posterior, or global topographical distribution. While anterior ASSs seem to depend mostly on generators within the ventrobasal thalamic nucleus, posterior ASSs appear to be largely initiated by the TRN [20]. This nucleus is also recognized as the main pacemaker for the generation of fast ASSs in humans as detailed below.

### 2.1. Generation and Origin of Adult Sleep Spindles

The generation of fast ASSs with thalamic origin has been divided into three stages: (i) initiation, (ii) propagation, and (iii) termination.

(i) In line with their ability to initiate rhythmic discharges, neurons in the TRN are the main pacemakers of ASS activity [31]. Reduced excitatory drive from cortical and subcortical afferents, present at the onset of NREM sleep, allows progressive hyperpolarization of TRN cells and a shift of their resting potential to values <−60 mV [32, 33]. At this hyperpolarized membrane potential, selective depolarization of TRN cells by cortical afferents leads to activation of low-voltage gated T-type Ca^2+^ channels that cause dendritic Ca^2+^ accumulation. The rise in Ca^2+^ triggers Ca^2+^-dependent small-conductance type 2K^+^ channels (SK2). As a consequence, burst afterhyperpolarizations are induced and lead to temporal inactivation of earlier triggered T-type channels [34–39]. Such alternations of depolarized and hyperpolarized states in TRN cells shape the typical spindle oscillations.
(ii) Rapid changes of ion concentrations are not sufficient to keep up ASS activity. Additional cellular interplay maintains and particularly propagates ASSs. TRN cells form dense inhibitory connections with thalamocortical (TC) cells in the dorsal thalamus [40]. In reaction to synchronized inhibition by TRN cells, TC cells show paradoxical activation and fire postinhibitory rebound bursts [41, 42]. Excitatory back-projections from TC to TRN cells establish a self-maintaining excitation-inhibition cycle that enables stronger network synchronization and progressive recruitment of further thalamic cells [37]. In addition, coupling of TRN cells by gap junctions facilitates synchronization of the reticular network activity [43, 44]. In line with their nomenclature, TC cells project not only to reticular neurons, but also to different areas of the cortex, primarily targeting fast-spiking interneurons in layer IV [45–47]. Subsequent propagation between cortical layers amplifies the oscillatory activity, whereas deeper cortical layers provide feedback to TC and TRN cells to maintain the thalamic entrainment [48]. The importance of the cortex and the corticothalamic feedback for ASS synchronization and amplification is reflected by reduced ASS synchrony and phase locking after cortical depression and locally restricted synchronization after decortication [49, 50].
(iii) To prevent unrestrained excitation and concomitant development of epileptic seizures, several mechanisms control and terminate ASS activity. First, GABA_A_-receptor-mediated lateral inhibition between TRN cells prevents the occurrence of hypersynchrony in the thalamocortical network [42]. Second, the strong accumulation of Ca^2+^ in dendrites of TRN cells activates the sarco- (endo-) plasmatic reticulum Ca^2+^ ATPase (SERCA) that pumps Ca^2+^ back into the cellular stores and interrupts the T-SK2 channel interaction [34, 51]. Another effect of the Ca^2+^ accumulation is the persistent upregulation of *I*_*h*_ in TC cells. This upregulation is caused by a Ca^2+^-induced Ca^2+^ release and a facilitated binding of cAMP to open hyperpolarization-activated, cyclic-nucleotide-gated (HCN) channels. The resulting afterdepolarization prevents the generation of further rebound bursts in TC cells [52, 53]. Finally, reduced synchronization and phase locking of the thalamus and cortex diminishes rebound bursts in TC cells and stops further recurrent entrainment of the network [54].

### 2.2. Adult Sleep Spindles Boost Plasticity

The mechanisms involved in the initiation, propagation, and termination of ASSs control the synaptic plasticity processes in the corresponding adult networks. Strong and fast increase of local intracellular Ca^2+^ concentration, triggered by NMDA receptor activation after voltage-dependent release of the Mg^2+^ block and opening of voltage gated Ca^2+^ channels (VGCC), activates postsynaptic signaling cascades involving protein kinases, such as PKA and Ca^2+^/calmodulin-dependent protein kinase II (CaMKII), which represent key players for long-term potentiation (LTP) [55–58]. CaMKII facilitates synaptic potentiation by phosphorylation of AMPA receptors and augmentation of GluR1-containing AMPA receptors at the postsynaptic density [59]. Modelling of plasticity processes in the hippocampus supports a correlation of activated CaMKII with the occurrence of cortical ASSs at the transition between sleep stages [60]. Of note, ASSs occurring outside this transition period did not correlate with the hippocampal CaMKII level.

While strong and fast Ca^2+^ increase contributes to LTP induction, small and long-lasting Ca^2+^ has been shown to generate protein phosphatase dependent long-term depression (LTD) [61]. Furthermore, Ca^2+^-signaling is important for spike-timing dependent plasticity (STDP) [62]. Dendritic Ca^2+^ influx through VGCC caused by back-propagating action potentials [63] leads to a supralinear increase of local intracellular Ca^2+^ concentrations and promotes LTP [64–66]. Short-term potentiation and LTP were induced by repetitive pre- and postsynaptic stimulation of cortical layer V pyramidal cells with ASS-associated spike trains, whereas presynaptic stimulation alone led to LTD [67]. Shuffling and mirroring of the ASS spike train used for stimulation failed to induce synaptic potentiation. This indicates that the temporal order of the recorded ASS spike train intervals was ideal to evoke synaptic changes.

### 2.3. Adult Sleep Spindles in Memory Functions and Network Plasticity

ASSs present during specific sleep phases have been proposed to be beneficial for several forms of memory including declarative [68, 69], procedural [70], and emotional memory [71]. The density of ASSs, especially, has been correlated with the performance in memory retrieval after sleep [72, 73]. However, the exact functions of different sleep stages in relationship with corresponding rhythmic neuronal activity are still poorly understood [3, 5]. Natural sleep in mammals is comprised of about 20% rapid eye movement (REM) and 80% NREM sleep. NREM sleep can be further divided into several stages from drowsiness (N1) over light sleep (N2) to deep, restorative SWS (N3) [11]. EEG and local field potential measurements showed that distinct activity patterns characterize these different stages of natural sleep. On the one hand, REM sleep is characterized by the occurrence of ponto-geniculo-occipital (PGO) waves and hippocampal theta oscillations. On the other hand, cortical slow oscillations, thalamocortical spindle activity, and hippocampal sharp-wave ripples are prominent during NREM sleep [32, 74]. Such rhythmic network activity is supposed to coordinate neuronal activity and to facilitate the integration of information based on the synchrony of convergent inputs, as well as the selection of inputs depending on their timing [1, 2].

The slow oscillations seen in SWS synchronize over large cortical areas and produce alternating depolarized UP and hyperpolarized DOWN states [75]. As common input to neuronal ensembles is relevant for induction of synaptic plasticity, the alternation of UP and DOWN states and, accordingly, long-range network synchronization is thought to provide temporal windows for memory consolidation processes [56, 76]. Furthermore, ASSs might shape memory-related plasticity during NREM sleep on subcellular level. For example, formation of spines on specific dendritic branches of layer V pyramidal neurons in the motor cortex during sleep has been recently observed following a motor learning task [77]. Branch-specific spine formation was shown to depend on reactivation of task-specific synapses and increase of somatic Ca^2+^ levels during subsequent NREM sleep, but not REM sleep [77]. Taking into account the role of ASSs in control of cellular Ca^2+^ concentrations, it is highly likely that ASS activity is involved in the branch-specific spine formation.

However, not all newly encoded memory traces become consolidated during sleep. Sleep favors memories expected to yield future rewards or being relevant for survival. Therefore, the question arises, which mechanisms are responsible for the selection of information for later consolidation? Two major hypotheses have been proposed.

The hypothesis of active system consolidation claims that the active transfer of information is encoded and stored in the neocortex and hippocampus during wakefulness but transferred into cortical long-term memory stores at sleep [78]. To enable this transfer, fast ASSs generated by the thalamus build a unitary complex with cortical slow oscillations and hippocampal sharp-wave ripples (80–200 Hz) [79, 80]. Slow oscillations appear to synchronize the occurrence of ASSs and the repeated accelerated reactivation of memory representations in form of hippocampal ripples during cortical UP states. In both humans and rodents, a clear phase locking between cortical ASS activity and hippocampal ripples can be observed with ripples occurring within the troughs around the peak of ASS activity [81–83]. This suggests the presence of a feedback loop that would enable a precisely timed bidirectional information transfer between cortex and hippocampus promoting synaptic plasticity and consolidation of memory [22].

The synaptic homeostasis hypothesis proposes a different mechanism and suggests that a global downscaling of synapses during sleep counterbalances their strengthening during encoding of new information at wakefulness. The concomitant synaptic potentiation and increase in firing during wakefulness puts the brain under a higher energy demand than continuously sustainable [84, 85]. The resulting progressive increase of glutamate in the extrasynaptic space would lead on the long run to cell intoxication and death [86]. Therefore, a process that resets the catabolic demand and reduces the stress within the brain seems to be mandatory. Likewise, an increasing amount of potentiated synapses reduces the signal-to-noise ratio within networks and prevents flexible responses to changes in the environment [87]. Depending on the actual electric state at the postsynapse recurrent bursts during episodes of ASSs can enable global downscaling of synaptic strength via long-term depression (LTD) [67, 88]. Apart from the attenuation of energy consumption, this global downscaling would also contribute to the emergence of information still concealed by less relevant information during wakefulness [5].

These two hypotheses might complement each other and share mechanisms relevant for memory consolidation. For example, both hypotheses need meaningful strategies to enable the transformation of labile encoded memory traces into long-lasting information during sleep. Recently, Heib and colleagues described a close relationship of event-related increase in hippocampal theta activity during wakefulness and the amount of fast ASS activity in subsequent sleep [89]. Theta oscillations are important for attentional shifts, top-down control of gamma oscillations, and consecutive memory formation [2, 90, 91]. Their correlation with ASS activity allows speculating about a participation of theta activity in the selection and tagging of meaningful memory traces [89]. In support of this, correlation of theta activity and subsequent increase in ASSs during SWS was also shown for theta oscillations occurring in REM sleep [92]. Similar to the slow oscillations during SWS, theta activity might enable the information transfer between hippocampus and cortex either during performance of a task or during theta replay in REM sleep and prepare selected synapses for further consolidation in SWS episodes.

### 2.4. Adult Sleep Spindle Pathologies

Deeper understanding of ASS function could be achieved by the investigation of spindle-associated pathologic states. A wealth of studies documents the link between abnormal ASSs and disabilities in neurological disorders. For example, altered ASSs have been related to hypersynchronous activity between thalamus and cortex in different forms of epilepsy [93–95]. Moreover, the occurrence of ASSs is dramatically perturbed in neuropsychiatric disorders, such as schizophrenia or depression [96–98].

In young and adult schizophrenia patients a widespread reduction of ASS occurrence and power has been detected over centroparietal, prefrontal, and temporal areas of the cortex [99, 100]. The overall intelligence of patients is not affected [101], yet lower ASS occurrence correlates with abnormal memory consolidation and the severity of positive symptoms, in particular of auditory hallucinations [98, 101, 102]. The poorer memory consolidation in schizophrenia patients has been correlated with the reduced volume of the left mediodorsal thalamus, including the spindle pacemaker TRN [103–106].

Even if less consistent as for schizophrenia, changes in ASS activity have also been reported for patients with major depression [107]. High-risk individuals and age-matched early-onset depression patients showed reduced ASS density when compared to controls. This decrease was more pronounced in females [108]. With increasing age, the shortage of spindle activity is overcompensated, the ASSs being more frequent in adult female patients when compared to healthy controls [107, 108]. While it cannot be fully excluded that the developmental switch results from methodological differences between studies, it is highly likely that extensive remodeling of brain circuits or hormonal changes during adolescence account for the age-dependent transition from shortage to surge. Males showed milder alterations in ASSs.

The structural and functional substrates of abnormal ASS activity in disease are poorly understood. Brain connectivity in regions relevant for spindle generation and glutamate signaling is disturbed in schizophrenia patients [109–112]. Similarly, in a Disrupted-In-Schizophrenia 1 (DISC1) mouse model of mental illness ^14^C2-DG imaging revealed pronounced hypometabolism in frontal and hippocampal regions as well as in the TRN. The observed abnormal functional communication between brain areas was accompanied by reduced glutamate release probability [113]. A model with transient interruption of DISC1 signaling showed a loss of plastic compensatory mechanisms. After whisker deprivation during early development, healthy mice usually react with a compensatory expansion of the whisker-corresponding domain into surrounding cortical barrels [114]. This structural modification was absent after transient DISC1 interruption. At adult age, these mice showed a complete absence of intercolumnar LTP and LTD [115]. The impairment of essential mechanisms for learning, like LTP and LTD, might prevent a proper encoding of new information during wakefulness and reduce ASS generation during sleep (see the previously discussed role of ASSs in memory formation). Moreover, studies in calcineurin knockout [116] and dominant-negative DISC1 [117] mouse models of schizophrenia showed a strong increase in power and occurrence of hippocampal sharp-wave ripples. This increase was accompanied by the loss of ripple replay function. Since ripple replay has been proposed as coordinator of cortical ASS activity [83], memory consolidation during SWS might be disturbed in these mice.

In summary, dysfunction of ASS activity seems to be a promising predictive marker for certain neurologic and neuropsychiatric conditions. Future investigations need to strengthen the link between abnormal patterns of activity and disease and unravel associated structural and functional modifications at cellular level. The knowledge gain of such studies will enable the development of therapeutic strategies aiming at improving the cognitive outcome of patients.

## 3. Neonatal Spindle Burst Oscillations

Oscillatory rhythms are not an exclusive hallmark of the adult brain but emerge already early in life. They have been characterized in EEG recordings from premature human infants [118, 119]. However, technical and ethical limitations precluded the elucidation of mechanisms underlying early oscillatory rhythms in humans. Since rodents are altricial and the stage of their brain development at birth corresponds to the second gestational trimester in humans, they represent an ideal animal model for the investigation of early patterns of activity [120, 121], which are highly reminiscent to those recorded in human preterm babies [13, 122, 123].

Early neuronal activity has a discontinuous structure with alternating periods of oscillatory discharges (2–30 s duration) and network silence [14, 15, 124, 125]. With ongoing maturation, the discontinuous activity is progressively replaced by adult-like continuous discharges. During neonatal development (i.e., first-second postnatal week) a large diversity of discontinuous oscillations have been described in the rodent cortex. The most common pattern is the NSBs [14, 124]. These oscillations have a duration of 1–3 s and a frequency of 7–10 Hz. They can be superimposed with faster beta/gamma activity and high frequency oscillations (HFOs) [126, 127] and are then classified as nested gamma spindle bursts (NGs). For visual areas, slow activity transients of <0.5 Hz, also known as delta waves, seem to coordinate NSBs [128], whereas long-oscillations (20–110 s duration) have been characterized in the primary somatosensory cortex [125]. Ca^2+^ imaging revealed similar activity patterns spreading along the posterior-anterior cortical axis [129, 130]. Brief periods of early gamma oscillations also occur independent of spindles in cortical [125], thalamic [131], and hippocampal networks [132]. Despite the diverse nomenclature, it is likely that the early network oscillations share similar mechanisms of generation.

Synchronization and coupling of neuronal networks in oscillatory rhythms early during development organize the communication of spatially distributed neuronal subsets. They increase the probability of cooccurring pre- and postsynaptic activity and by these means control synaptic plasticity. Each burst recruits a different set of synapses and enforces the potentiation of parallel activated neighboring synapses and dendritic clustering [133–135]. In the following we will focus on the most dominant pattern of discontinuous activity, the NSBs.

### 3.1. Generation and Origin of Neonatal Spindle Bursts in Sensory Systems

During the last 10–15 years substantial effort has been made to elucidate the mechanisms of NSB generation. Stimulation of the optical nerve, mechanical touch of the limbs, or whisker stimulation reliably triggered NSBs in primary sensory cortices [14, 15, 124]. Correspondingly, interruption of peripheral sensory inputs by brain stem lesion [124], pharmacological blockade [12], or removal of the retina [14] leads to a strong reduction in the occurrence of NSBs. These studies reveal the importance of the sensory periphery/external stimuli for the emergence of NSBs during development. They equally demonstrate that NSBs partially depend on the activity of intrinsic pacemakers [136].

In neonatal rodents an important region for the amplification and integration of information from the periphery turns out to be the subplate. Subplate cells originate in the ventricular and subventricular zone as well as the medial ganglionic eminence. At early developmental stage, they show adult neuronal characteristics with a heterogeneous morphology and neurotransmitter profile as well as dense connectivity [137, 138]. Subplate cells form a transiently expressed layer located between the intermediate zone and the cortical plate [139, 140]. They guide axons from subcortical structures, such as thalamus, to their appropriate targets in the developing neocortex and are ideally positioned to shape neocortical plasticity [141–143]. The early networks, which are organized by subplate neurons, are driven by thalamocortical projections and modulated by cholinergic afferents from the basal forebrain. By these means, spatially confined synchrony (e.g., barrels in the somatosensory cortex, ocular dominance columns in the visual cortex of higher mammals) is established [136, 144–146]. NSBs, which play an important role in this synchronization, emerge within the thalamocortical networks at neonatal age [124, 147]. Removal of subplate neurons in rats at postnatal days (P) 0-1 prevents the emergence of spontaneous and evoked NSBs in the somatosensory cortex at P7–10 together with a weakening of thalamocortical connectivity [16].

The relay of information from the periphery via thalamic nuclei and the subplate to specific areas of the cortex appears to follow a universal scheme and is similar for different sensory modalities. In the primary somatosensory cortex of P0-1 rats, whisker stimulation induced gamma activity in the ventral posteromedial nucleus of the thalamus (VPM) followed by shortly delayed NSBs [136]. Inactivation of the VPM with electrolytic lesion almost abolished NSBs [136]. For the visual system, Mooney and colleagues showed that spontaneous retinal activity elicits bursts in the lateral geniculate nucleus (LGN) of the thalamus. Burst activity in the LGN was suppressed after pharmacological blockade or cut of the optic nerve [148]. In addition, blocking action potential propagation in the optic nerve by TTX injection led to a twofold decrease of the NSB occurrence in the visual cortex [14]. In the auditory system, inner hair cells in the cochlea of neonatal rats (P7) generate discrete bursts of action potentials that propagate along central auditory pathways already before hearing onset [149, 150]. This activity was shown to be crucial for the establishment of precise tonotopy in auditory nuclei, for example, lateral superior olive [151]. Although a clear link of these bursts to activity in higher structures of the auditory pathway is missing, it has been demonstrated that subplate neurons receive input from the medial geniculate nucleus of the auditory thalamic nucleus from P2 on. These subplate cells provide excitatory input to layer IV neurons in the auditory cortex [152]. With the medial geniculate nucleus lying upstream of the lateral superior olive, it is likely that the transfer of information in the auditory system during early development follows similar principles as for visual and somatosensory systems. Altogether, these findings suggest the importance of peripheral input and corticothalamic connectivity for the generation of NSBs in various sensory networks.

Another interesting aspect in the generation of NSBs is their early dependence on neuromodulatory inputs. For example, the cholinergic drive from the basal forebrain profoundly influences the NSB activity at neonatal age. While electrical stimulation of the basal forebrain increases the incidence of NSBs, selective immunotoxic lesion of its cholinergic neurons with antibody-conjugated saporin and pharmacological blockade of cortical muscarinic acetylcholine receptors lead to substantial reduction of NSB occurrence [153]. In contrast, blockade of acetylcholine esterase promoted NSB generation. These data support a facilitating action of the cholinergic system on NSBs. One possible substrate of this function is the cholinergic action on subplate neurons. Ca^2+^ transients induced by muscarine application show high synchronicity in the subplate region, while transients in the cortical plate appear less frequent and more random [143].

NSBs in the sensory cortices are significantly modulated by interhemispheric communication. After callosotomy at P1–6, the presence of spontaneous NSBs in both hemispheres is doubled, indicating that projections via the corpus callosum inhibit developmental activity patterns during the first postnatal week [154]. In contrast, callosotomy at P7–15 does not alter the rate of spontaneous NSBs [155]. Moreover, callosotomy reduced the occurrence of NSBs evoked by forepaw stimulation in the somatosensory cortex during defined developmental periods (i.e., P1–6). It can be hypothesized that a period critical for spindle related plasticity spans the time from birth to P7. Remarkably, this developmental period coincides with an increase of GABAergic and glutamatergic presynaptic terminals in the deeper layers of the somatosensory cortex [155].

The mechanisms generating early network oscillations vary during the course of development [156].* In vivo* investigations have shown that glutamatergic inputs are critical for the emergence of NSBs [15, 16, 157, 158]. The slow delta components of NSBs are reliant on both NMDA and AMPA receptors whereas the faster spindle component mainly depends on AMPA receptors [158]. Pharmacological blockade of AMPA receptors using CNQX completely and reversibly blocked the occurrence of spontaneous spindle bursts in S1 indicating the importance of the glutamatergic inputs for the generation of NSBs [15]. As previously mentioned ablation of subplate neurons weakens the thalamocortical connectivity and reduces the occurrence of NSBs suggesting that the glutamatergic inputs important for NSBs are of thalamic origin [16]. Knockout of the NR1 NMDA receptor subunit in the ventrobasal thalamic nucleus resulted in miswiring of the barrel cortex and behavioral deficits supporting a contribution of NMDA receptors to STDP already during early development [157]. Additionally, electrical communication through gap junctions contributes to the generation of NSBs. Inhibition of gap junctions was followed by reduction or abolishment of spindle oscillations both* in vitro* [159] and* in vivo* [125]. These data indicate that an early gap junction syncytium acts as template for later cortical topography [160] and represents an efficient mechanism of communication at a developmental time point of synaptic immaturity [161, 162]. However, due to the side-effects of many gap junction blockers, the contribution of electrical communication to early spindle activity remains a matter of debate and findings are sometimes contradictory. For example,* in vivo* blockade of gap junctions at P1–P3 has been shown to cause an increase in the occurrence of spindle bursts [15].

The generation of NSBs is additionally influenced by GABAergic neurotransmission. During the embryonic stage and the first postnatal week an increased chloride accumulation/extrusion ratio due to high NKCC1 activity and low KCC2 expression causes a depolarizing action of GABA in immature neurons [163, 164]. Early depolarizing GABAergic activity is linked to critical period plasticity in the visual cortex [165]. Interference with early depolarizing GABA_A_ receptor signaling was shown to persistently reduce the formation of AMPA receptors. Furthermore, the tight interaction of GABA_A_ and NMDA receptors controls the activation of silent synapses [166]. Blockade of NKCC1 was shown to cause a negative shift in the GABA_A_ reversal potential but did not affect the occurrence and properties of NSBs [15]. However, GABA_A_ receptor-mediated depolarization has been recently shown to exert an inhibitory control on network activity* in vivo* [167]. Such early GABAergic inhibition is in line with enhanced NSB activity after GABA receptor blockade, reduced activity after positive modulation of GABA_A_ receptors [15], and the confinement of early network oscillations by GABAergic surround inhibition [15, 168, 169]. The precise actions of GABA may not only depend on the maturation level of an individual neuron but also on the timing of GABAergic and glutamatergic inputs. Recently, it was shown* in vitro* for adult born neurons in the hippocampus that weak GABAergic input is beneficial for neuronal excitation and strong GABAergic input leads to shunting inhibition [170]. Such a mechanism would contribute to the spatial confinement of NSBs in local cortical networks. Around P12 an increase in KCC2 expression and reduction in NKCC1 activity in cortical neurons shifts the GABAergic transmission to the “classical” hyperpolarizing function [171]. Of note, this switch does not occur at the same time point in the entire cortex, but rather depends on the maturation timeline of individual cortical areas. Even within the same area, the hyperpolarizing action of GABA starts in more mature deeper cortical layers before it reaches the upper cortical layers [172]. However, in general, this time point coincides with the disappearance of NSBs during the end of the second postnatal week. NSBs are gradually replaced by adult-like ongoing oscillations within different frequency bands related to behavioral states. Early GABAergic transmission is also relevant to shape emerging cortical networks. During the first postnatal week layer 5/6 somatostatin interneurons receive transient innervation from the thalamus. This innervation supports the development of parvalbumin interneuron driven perisomatic inhibition of pyramidal neurons, which is crucial for the generation of fast rhythmic activity in the adult cortex [173]. The role of NSBs for the development of this network needs further investigation.

### 3.2. Generation and Origin of Neonatal Spindle Bursts in Limbic Systems

NSBs are not restricted to sensory cortical areas but also synchronize limbic structures [126, 174]. In line with the developmental delay of the prefrontal cortex (PFC) compared to the sensory areas, NSBs are absent at birth and firstly detected in the PFC at P3 [14, 126]. With ongoing maturation, the power of NSBs augments. In the cingulate subdivision of the medial PFC, NSBs contain little or no gamma activity. In contrast, frequent NGs have been detected in the prelimbic subdivision (PL) of the PFC [126]. NSBs synchronize the activity within prefrontal layers, whereas the faster gamma components of NGs synchronize the coupling between different layers [127]. The distinct activity patterns in prefrontal subdivisions might reflect an adapted maturation in regard to their later functionality.

Similar to the importance of thalamocortical networks for the generation of NSBs in sensory areas, the hippocampal-prefrontal networks appear to be mandatory for the emergence of limbic NSBs. The drive and temporal coordination within these networks is provided by theta oscillations from the hippocampal CA1 area. These are relayed to pyramidal neurons of layer V in the PFC via glutamatergic projections, which in turn trigger local beta/gamma activity in cortical networks [126, 175, 176]. Excitotoxic lesion of the hippocampus led to substantial diminishment of prelimbic oscillatory activity [126]. First correlative evidence suggests that the coherent activity within neonatal prefrontal-hippocampal networks is critical for juvenile cognitive abilities, such as recognition memory [177].

Similar to oscillatory patterns in sensory cortices, NSBs in limbic areas depend on cholinergic modulation. Cholinergic projections from the basal forebrain selectively target interneurons in the neonatal PL [174]. Neurotoxic lesion of cholinergic nuclei in the basal forebrain profoundly affected the activity within prefrontal-hippocampal networks by increasing the occurrence of hippocampal theta oscillations and the NSB amplitude in the PFC [174]. Thus, NSBs in sensory and limbic cortices share similar properties and mechanisms of generation. While an external drive (e.g., activation of sensory periphery, hippocampus) is mandatory, NSBs additionally emerge from the activation of local circuits and are strongly modulated by subcortical inputs.

## 4. Comparison of Adult Sleep Spindles and Neonatal Spindle Bursts

**Figure 1:**
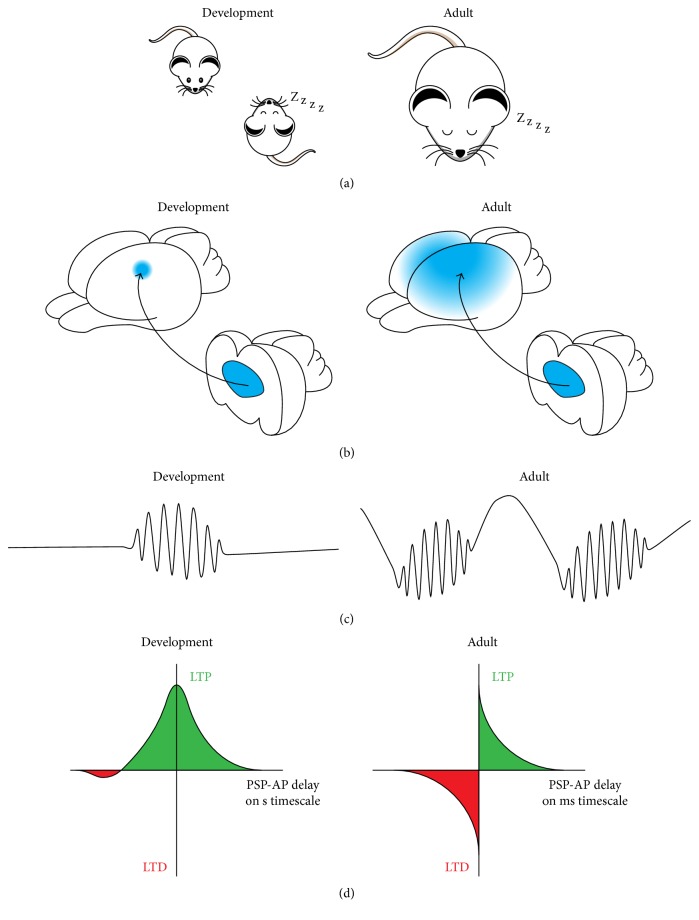
Spindle activity in development and adult sleep. (a) NSBs occur independent of the behavioral state (left), whereas ASSs are confined to SWS (right). (b) Thalamocortical activity locally synchronizes the developing networks (left), whereas ASSs entrain large cortical areas (right). (c) NSBs represent discontinuous patterns of oscillatory activity during early development (left). ASSs are embedded in slow oscillations (right). (d) Synchronized activity during development leads to synaptic potentiation (left). Precise timing of synaptic inputs and action potentials controls synaptic potentiation and depression in mature brains (right).

Transient bursts of network oscillations are present in cortical networks during early development and at adulthood. They are generated, at least in part, within thalamocortical and hippocampocortical circuits. Several features distinguish NSBs and ASSs (Figure 1). First, NSBs are not restricted to sleep but occur during all behavioral states [124]. Second, NSBs synchronize relatively small cortical patches [124, 125], whereas ASSs spread over large cortical areas. Third, NSBs occur spontaneously at irregular intervals [12, 14, 124], whereas ASSs are generated more regularly.

While synaptic plasticity is present in the brain throughout the whole lifespan, mechanistic differences were described for the developing and the adult brain. In general, synaptic potentiation is more prominent during development and gradually changes towards a balanced potentiation and depression in the mature brain. This phenomenon is probably related to a switch in the mechanisms that mediate LTP. Before P10 the induction of LTP in the rodent hippocampus was shown to be PKA and GluR4-dependent [178–180] and could be induced by random neuronal activity [181]. From P12 on LTP was driven by activation of CaMKII and GluR1 and plasticity started to follow precise STDP rules, including LTD [182, 183]. In the barrel cortex the mechanisms mediating LTP are similar, yet the switch from PKA and GluR4 dependence to CaMKII and GluR1 dependence occurs at P13 [184, 185]. A bias towards potentiation based on the timing of bursts over a second-long time window was also described in the immature thalamus [186].

The switch in plasticity mechanisms is further reflected by the massive increase in the number of synapses and connections during the early postnatal period. During the third postnatal week synaptic pruning causes profound network refinement [187–189]. In other words, a rough connectivity scheme at early development is established long before the emergence of precise topographic maps. Consequently, the occurring plastic processes are constantly modulated by changes in molecular expression and ongoing network activity. While neonatal and adult spindle oscillations demonstrate similarities in shape, frequency distribution, and origin, they are faced with different plasticity conditions and therefore differentially modulate brain circuits.

The knowledge on the role of spindle activity during early development and at adulthood is still sparse. It is accepted that early network oscillations promote the maturation of cortical structure and function, yet reliable causal evidence is still missing. Repetitive coactivation of specific networks during NSBs would create optimal conditions to strengthen and refine synaptic connections [134, 185, 186]. ASSs are implicated in memory consolidation and homeostatic scaling of synaptic strengths. They may coordinate the communication of specific networks in faster frequencies to orchestrate the interplay of synaptic depression and potentiation dependent on the timing of pre- and postsynaptic activity on a millisecond timescale. Thus, NSBs may provide time windows of synchronous activity necessary for the refinement of circuitry, whereas ASSs may help to adapt the mature network to integrate recently acquired information.

## 5. Conclusions and Perspectives

ASSs and NSBs represent distinct patterns of network synchronization in the adult and developing brain. While ASSs support memory consolidation through synchronous activation of large cortical areas, NSBs coordinate the maturation of local neocortical networks. Both patterns coordinate activity in sensory and limbic systems and modulate local plasticity critical for network refinement. Causal links from specific cellular mechanisms to oscillatory activity need to be established. Knowledge about NSB-related plasticity, especially, is still sparse. Further studies need to elucidate to what extent disturbed NSB activity during cortical maturation affects the pathological changes observed for ASSs, for example, in schizophrenia and depression. This would allow developing new therapeutic approaches to prevent manifestation of neurodevelopmental diseases.
